# Diffusion and Swelling Measurements in Pharmaceutical Powder Compacts Using Terahertz Pulsed Imaging

**DOI:** 10.1002/jps.24376

**Published:** 2015-02-02

**Authors:** Samy Yassin, Ke Su, Hungyen Lin, Lynn F Gladden, J Axel Zeitler

**Affiliations:** Department of Chemical Engineering and Biotechnology, University of CambridgePembroke Street, Cambridge, CB2 3RA, UK

**Keywords:** terahertz pulsed imaging, diffusion, porosity, swelling, solid dosage forms, imaging methods, hydration, oral drug delivery, microstructure, X-ray micro computed tomography

## Abstract

Tablet dissolution is strongly affected by swelling and solvent penetration into its matrix. A terahertz-pulsed imaging (TPI) technique, in reflection mode, is introduced as a new tool to measure one-dimensional swelling and solvent ingress in flat-faced pharmaceutical compacts exposed to dissolution medium from one face of the tablet. The technique was demonstrated on three tableting excipients: hydroxypropylmethyl cellulose (HPMC), Eudragit RSPO, and lactose. Upon contact with water, HPMC initially shrinks to up to 13% of its original thickness before undergoing expansion. HPMC and lactose were shown to expand to up to 20% and 47% of their original size in 24 h and 13 min, respectively, whereas Eudragit does not undergo dimensional change. The TPI technique was used to measure the ingress of water into HPMC tablets over a period of 24 h and it was observed that water penetrates into the tablet by anomalous diffusion. X-ray microtomography was used to measure tablet porosity alongside helium pycnometry and was linked to the results obtained by TPI. Our results highlight a new application area of TPI in the pharmaceutical sciences that could be of interest in the development and quality testing of advanced drug delivery systems as well as immediate release formulations. © 2015 Wiley Periodicals, Inc. and the American Pharmacists Association J Pharm Sci 104:1658–1667, 2015

## Introduction

Solid dosage forms are used to deliver active pharmaceutical ingredients (APIs) to treat a range of conditions, and advanced drug delivery formulations can be used to adjust the drug release over short, long, or sustained periods of time at a specified rate. In addition, solid dosage forms can be tailored to work in specific parts of the gastrointestinal tract. There are numerous methods for controlling the release of API with immediate-release formulations and sustained-release matrix formulations forming the focus of this investigation.^1^

For a range of matrix formulations, it has been demonstrated that the interaction of the tablet with its surroundings can be described by the so-called shrinking core mechanism and variants thereof.^2^ The shrinking core mechanism model describes diffusion-controlled processes such as the interaction of the dissolution medium with the solid and its ensuing physical conversions. Mathematically, this process is described to occur layer by layer until the drug release is complete,^2^ and although it provides very good estimates for some formulations, it is not suited for all formulations. The shrinking core mechanism can establish whether diffusion or a reaction (chemical or physical) is the rate-controlling mechanism; this is traditionally used on particles with a fixed volume; therefore, numerous assumptions have to be taken regarding the tablet and intraparticular geometries over the experimental procedure.^2^ More sophisticated analytical procedures to investigate the dissolution process of tablets open the potential to devise better models that can be used to predict and design tablet performance enabling formulators to move from a largely empirical development process to a refined design problem.

The changes, that take place in the dosage form upon contact with the dissolution medium on the molecular and microstructural level, are complex and still not fully understood. For example, the release of API from a swelling polymer matrix formulation such as hydroxypropylmethyl cellulose (HPMC) involves multiple steps on the molecular and microstructural level such as diffusion, capillary action, polymer unwrapping, gel layer formation, relaxation of compaction forces, among others.^3^ A number of studies have described models of the swelling and the diffusion of water into the tablet matrix as well as the subsequent drug release. In particular, magnetic resonance imaging (MRI) has been used noninvasively to provide a unique insight into the water and drug diffusion and swelling kinetics of HPMC formulations in detail. The rate of swelling in different grades of HPMC was found to be different when comparing the radial to axial swelling and it was shown that the tablets swell up to 300% of their original size in the radial direction and 150% in the axial direction.^4^ It was also shown that swelling is a result of two different physical processes: during the hydration of HPMC tablet matrices, gel layer formation occurs and at the same time relaxation of compaction forces takes place.^4^–^8^ The developing gel layer acts as a diffusion barrier that slows down further water uptake and in turn alters the swelling kinetics of the tablet.^9^ Further, MRI studies have also investigated this effect in commercial products that use HPMC as a swellable matrix former.^10^,^11^ In contrast to the nondestructive MRI analysis, a range of destructive methods have been used to analyze the dissolution process in HPMC matrices including thermo gravimetric analysis,^12^,^13^ near-infrared (NIR) spectroscopy, and infrared imaging,^14^–^16^ among other techniques. These techniques were used to assess the dissolution as well as the ingress of water into the tablet matrices.

Delalonde and Ruiz^17^ have shown that the swelling and diffusion kinetics are influenced by the rate of water penetrating the tablet matrix and the rate of air draining out. The changes in pressure prompted by this fluid transport, coupled with the movement of the solids within the matrix during the hydration can cause the tablet to contract during the initial phases of the experiments as observed previously.^4^ The rate of penetration of water into the tablet matrix, the dissolution, water uptake and release of API, as well as the swelling kinetics were found to be dependent on the porosity of the tablet matrices.^18^ Numerous techniques that work across a range of length scales can be used to measure porosity: X-ray microcomputed tomography (XμCT), helium pycnometry, mercury porosimetry, BET/BJH analysis, and others. Using a combination of these techniques, it is possible to extract both total porosity and pore size distribution. This information can then be used to look at how tablet voidage can affect the bulk transport mechanisms, which are known to be dependent on the size, shape, and molecular interaction of the pores.^19^ Further work has investigated the effect of compaction, polymer unwrapping, concentration of solvent, and excipient as well as dissolution medium on the diffusion process.^8^,^21^,^20^

A number of studies have demonstrated how experimental data and theoretical models can be combined to describe the diffusion and dissolution processes occurring within polymer-based matrix tablets.^3^,^7^,^8^,^23^,^22^ On the basis of empirical data, it is known that the rate of diffusion into and out of a tablet can often be described by a power law and the Peppas–Sahlin equation.^22^ Both the Peppas–Sahlin equation and the power law are used to differentiate between Fickian diffusion and case II relaxation,^22^ where Fickian transport is reliant on a concentration gradient and case II transport on an activity gradient.^24^ Anomalous diffusion is a mode of transport that contains both Fickian and case II characteristics, this mode of transport is also described using the power law.^22^

The theoretical models discussed above were developed and refined based on experimental data from MRI,^4^,^7^,^11^,^25^ NIR imaging,^26^ infrared imaging,^15^,^16^ and spectroscopy^27^ as well as other analytical tools. Terahertz imaging is a relatively recent technique for pharmaceutical analysis. Its ability to penetrate typical pharmaceutical tablets, the strong contrast it can resolve based on subtle changes in refractive index within structures and its high-time resolution make it an attractive technique to investigate the microstructure of tablets.^28^ For example, using terahertz time–domain spectroscopy, Bawuah et al.^29^,^30^ demonstrated a method to calculate the total porosity of a tablet in a transmission measurement. Terahertz-pulsed imaging (TPI) has been widely used to nondestructively measure the coating thickness distribution of coated pharmaceutical dosage forms.^31^,^32^ It has also been used to measure the surface density of uncoated tablets as well as tablet hardness.^33^ Previous research has demonstrated how MRI and TPI can be combined to quantify coating stability before and after accelerated storage conditions, and how changes in the coating stability affect the dissolution process.^11^ Here, we demonstrate that TPI can be used in a different context to resolve the penetration of water into a tablet with time, while simultaneously following how the dimensions of the tablet change.

## Materials and Method

### Materials

The samples were powder compacts of HPMC (K15m; ColorChem, Atlanta, Georgia), one of the most widely used commercial swelling hydrophilic polymers used for sustained drug release formulations^34^; Eudragit RSPO (Evonik, Hamburg, Germany), which forms nonswelling matrices that can also be used as a tablet matrix for sustained drug release; and lactose α-monohydrate (Meggle, Wasserburg, Germany), which is a soluble, filler, diluent, and bulking agent commonly found in immediate-release formulations. Tablets were compressed to a height of 1.5 mm with diameters of 10 or 13 mm depending on the compaction technique.

### Particle Size Measurements

The particles within the powders were measured using the Morphologi G3S, which utilizes optical microscopy (Malvern Instruments, Malvern, UK). The particle size distributions were calculated using the Morphologi v8.11 software (Malvern Instruments). The mean particle size, *D*_50_, of HPMC, Eudragit RSPO, and Lactose were 16.9, 8.6, and 22.1 μm, respectively.

### Compaction

**Figure 1:**
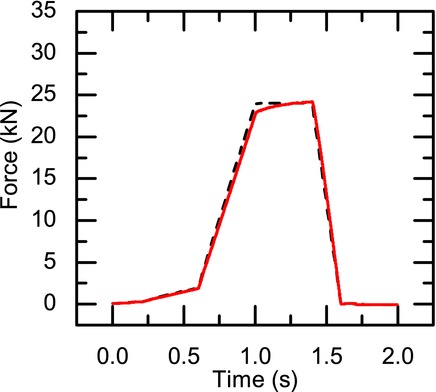
Compaction profile for the compression of Eudragit, lactose, and HPMC using the compaction simulator. The dashed black line corresponds to the readings from the lower punch and the red line shows the forces acting on the upper punch.

Tablets were compressed to 1.5 mm thickness, either by using a manual hydraulic press (Specac, Slough, UK) or a compaction simulator (Huxley Bertram, Cambridge, UK), to diameters of 13 and 10 mm, respectively. The compaction simulator was instrumented to record the forces throughout the compaction process. The amount of powder filled into the compression chamber was adjusted such that the tablets were compressed to a final height of 1.5 mm at 24 kN compaction force. In the compaction simulator, the compression of the tablets was carried out over a 2-s interval, where force was applied from the upper punch and the lower punch was kept stationary resulting in the compaction profile shown in Figure 1. Approximately 0.140 g of powder was required to compact tablets to height of 1.0 mm in the case of HPMC, and for both Eudragit RSPO and HPMC, approximately 0.250 g of powder is required to compact tablets to a height of 1.50 mm.

On the manual tablet press, tablets were compressed over a period of 5 min at a force of 24 kN. In contrast to the rapid compression that was achieved on the compaction simulator, this method leads to a more gradual compaction but through a much less well-controlled process.

As the tablets are being compacted using the same force but with two different tablet diameters, the tablets will be undergoing different compaction pressures of 306 and 181 MPa for the 10- and 13-mm diameter dies, respectively. This results in tablets of varying density and porosity. Tablets produced using a 10-mm die have a bulk density of 1150 ± 3.01 kg/m^3^, where the 13-mm die-produced tablets with a density of 1140 ± 9.46 kg/m^3^. Consequently, the resulting tablets have subtle variations in porosity, where tablets undergoing a larger applied pressure will have a pore size distribution with slightly smaller pores. For the set of tablets produced, the variation was found to be minor, but this change in porosity can potentially induce changes in dissolution performance.^35^

As the samples are being compacted over different time scales with compaction over a 5-min period versus a 2-s compaction process  stronger plastic deformation of the powder particles is possible during the long compaction period, whereas elastic deformation of the particles is likely to dominate during the fast process. This will further affect the particle size and shape distribution throughout the tablet matrix^36^ and hence the pore size distribution and microstructure of the compacts.^37^ It would be expected that the pore size distribution would decrease as a result of plastic deformation and such changes in microstructure would directly affect the disintegration performance.

### X-ray Microcomputed Tomography

The microstructure of the powder compacts was investigated using a Skyscan 1172 (Bruker, Kontich, Belgium) XμCT instrument. Using this technique, it is possible to nondestructively measure the pore volume distributions of the macropores (with diameters >4.55 μm) as well as analyzing the samples for the presence of any cracks.

Samples were placed into a clear Perspex tube and were held in place using spacers made from expanded polystyrene. The Perspex tube is rotated at 0.25° increments of rotation over 360°, and the measured shadow projections were used to reconstruct cross-sections of the samples in the Perspex holder. The Perspex tube and the air within the tube were used to calibrate the 8bit gray scale intensity during reconstruction between samples for subsequent threshold to ensure consistent binarization of the cross-sections between air and excipient. Reconstruction of the shadow projections was performed using the NRecon software (Skyscan, Version 1.6.9.3). The Dataviewer software (Skyscan, Version 1.4.4) was used to align the reconstructed datasets in *x*-, *y*-, and *z*-direction and then crop a 512 × 512 pixel region of interest traversing the center of the tablet from top to bottom. ImageJ version 1.47i^38^ was used to binarize the image and the BoneJ^39^ particle analyzer plug-in was used to calculate pore volumes.

### Helium Pycnometry

The density of the solid matrix of the tablets was obtained using the Accupyc 1330 Pycnometer (Micrometrics, Norcross, Georgia). Before undergoing pycnometry, samples were weighed. The 10-mm diameter samples are placed into a 1-cm^3^ sample cup, whereas the 13-mm tablets were placed into a 10-cm^3^ sample cup. The sample chamber was pressurized to 10 bar with helium and this was repeated 30 times, after which a density reading for the solid matrix was recorded (particle density, *D*_p_). Using these data, the density of the void space within the tablet was determined and the porosity was calculated.

### Diffusion Analysis Using TPI

**Figure 2:**
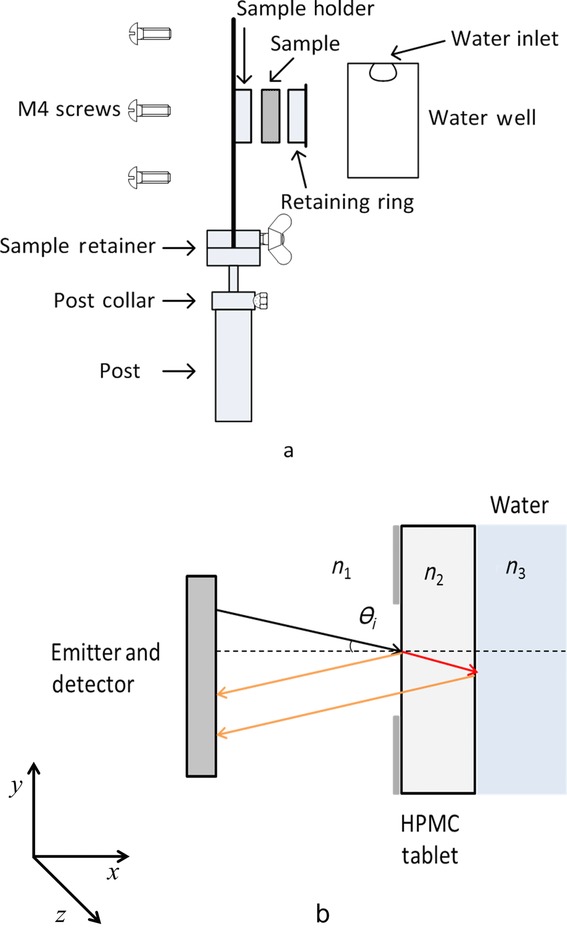
(a) Schematic diagram of the sample holder. (b) Schematic describing the analytical technique, where *n*1 represents the refractive index of air, *n*2 represents HPMC, *n*3 represents water, and *θi* is the incident angle of the terahertz beam.

The swelling and diffusion kinetics of dissolution medium in the tablets were measured using a modified TPI Imaga 2000 (Teraview Ltd., Cambridge, UK). A detailed description of the general principle behind the measurement technique was presented earlier.^40^ The TPI has a spectral range of 60 GHz to 3 THz, corresponding to wavelengths of 5.00 to 0.09 mm. High-resistivity silicon lenses and silicon probe optics focus the terahertz radiation to a diffraction-limited spot (200 μm diameter at a distance of 7 mm in front of the optics), where the sample is placed. A homemade sample holder (Fig. 2a) was used to fix the sample into place and expose it to water on one face while allowing for TPI reflection measurements through the opposite face.

In this setup, the entire surface of the radial band of the tablet is sealed from water, limiting the exposure from water to the back axial face of the tablet. By propagating a terahertz pulse into the tablet through its dry face, the one-dimensional diffusion of the dissolution medium can be studied in reflection mode, as shown in Figure 2b. This set up restricts the swelling of the axial surface at outer circumference to ensure the tablet is held in place; the majority of the front axial surface is free to swell as it is exposed to air. Like most polymers, HPMC is semitransparent to terahertz radiation and it is hence possible to assess physical changes within the tablet matrix that result in a change of its refractive index. At every point in the sample where the refractive index changes, a proportion of the radiation will be reflected, the amplitude of which will be proportional to the change in refractive index as defined by the Fresnel equations.^40^ In our measurements, 15 reflected waveforms are measured per second, and the waveforms are averaged depending on the duration of the diffusion process. For the experiments using sustained-release excipients, the diffusion process is monitored for a number of hours, whereas for lactose water breaches the front face of the tablet within 13 min, at which point the experiment is stopped. Therefore, 30 waveforms were averaged for the HPMC and Eudragit experiments and no averaging was performed for the lactose samples.

To facilitate the analysis, the waveforms were deconvolved mathematically to highlight the interfaces and remove noise.^41^ This was achieved by dividing the sample response by the reference and multiplying by the Fourier transform of the filter in the frequency domain as shown in Eq. (1),^42^ where FFT is the fast Fourier transform.


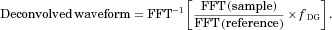
(1)

The reference waveforms used in these experiments refer to a reflection from the dry tablet before the tablet is exposed to water. The filter used was a double Gaussian filter that uses a high-frequency and low-frequency bandpass filter to remove background noise and this is mathematically described in Eq. (2),^41^ where *f*_DG_ denotes the double Gaussian filter, *t* is time, and HF and LF are the high-frequency and low-frequency limits, respectively. The mathematical product is then transformed back to the time domain using an inverse Fourier transform:


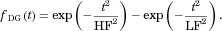
(2)

**Figure 3:**
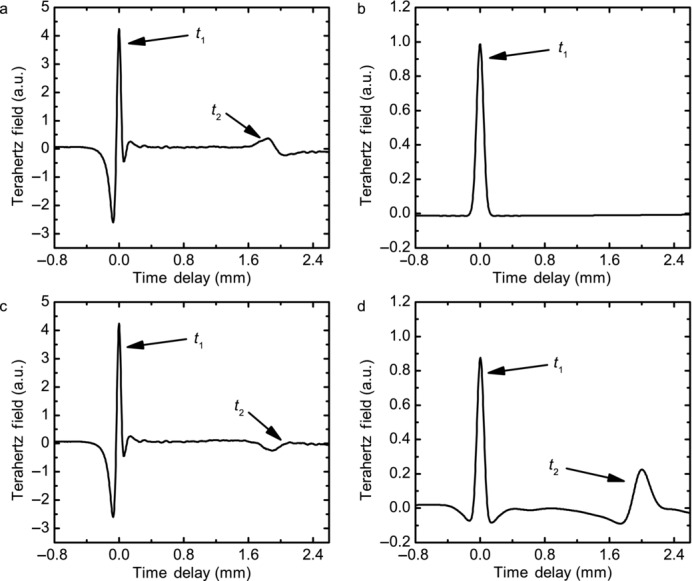
Raw and deconvolved waveforms. (a) and (c) Raw waveforms obtained using the TPI before and after water is in contact with the tablet, respectively. (b) and (d) Deconvolved waveforms corresponding to (a) and (c), respectively. The reflection from the front surface of the tablet is marked as *t*1, whereas *t*2 refers to the response from the back surface of the tablet.

Figure 3a is an example of a raw waveform produced from the dry tablet, Figure 3b shows an example of a waveform after deconvolution, Figure 3c is an example of a wetted tablet, and Figure 3d is its deconvolution. As in the raw waveform, each feature in the deconvolved waveform still originates from a change in refractive index but in this representation the interfaces are now easier to identify and to assign, as shown in Figure 3c: the first reflection *t*_1_ represents the front surface of the tablets, where the refractive index changes between air and the polymer. As air has a lower refractive index compared with the polymer, the peak is positive. The second peak *t*_2_ is a response arising from the change of refractive index between dry matrix former and water where the wet material has the higher refractive index because of the inherently higher refractive index of water *n* = 2.4^43^ compared with the porous polymer compact *n* < 1.5. This peak propagates over time, and represents a penetration front.

The intensity of these features is dependent on the amount of radiation that is reflected back to the emitter. This can be calculated using Fresnel's equation as shown in Eq. (3), where *r* is the reflectivity.


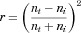
(3)

The distance of each refractive index interface from the emitter can be calculated using Eq. (4), where *n*_1_ is the refractive index of the sample, *c* is the speed of light in vacuum, and *t* is the time delay in seconds.


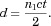
(4)

Both the deconvolved and raw waveforms are analyzed in two ways: the first analysis is tracking the movement of the front surface (*t*_1_) as water is entering the tablet at the back face, thus measuring the swelling kinetics of the tablets. The second analysis is to monitor and quantify the formation of any new features and their movements within the tablet matrix that can be assigned to the diffusion front.

## Results and Discussion

### Microstructure Analysis

**Figure 4:**
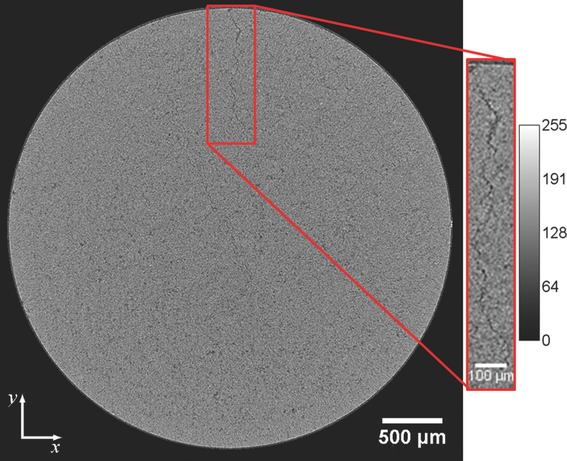
Cross-section at the center of a HPMC tablet produced using the compaction simulator. In this sample, a crack propagates into the tablet matrix in the radial direction and it extends from the top surface of the tablet to the bottom. The color bar shows the gray scale of the image in arbitrary units (a.u.).

**Figure 5:**
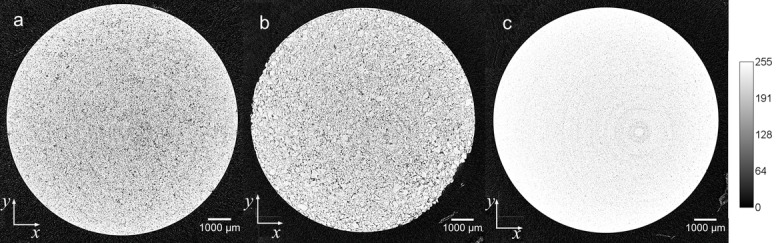
Reconstructed cross-sections of the XμCT images of (a) HPMC, (b) Eudragit, and (c) lactose; all prepared with the compaction simulator. The color bar shows the gray scale of the image in arbitrary units (a.u.).

The XμCT data were analyzed manually by inspecting each reconstructed *xy*-slice image of all samples to detect any structural defects such as cracks within the tablet (Figure 5). In addition to revealing cracks, the images clearly resolve grain patterns within the tablets, which were found to qualitatively differ between the three excipients as shown in Figure 4. Sufficiently large pore spaces to be resolved by XμCT were found, which were used to quantify the pore space in each tablet sample.

There are clear differences in the shapes of the grain in the compacts made from the three different excipients, in particular between HPMC and Eudragit. The grain patterns in the lactose tablets are much smaller and occur at a much lower frequency in comparison to Eudragit and HPMC; this is a result of the tablet reaching its solid limit. The grain boundaries can be a result of the particle shape prior to compaction as well as the mechanical properties of the particles.^44^ In Figure 4, the X-ray cross-sections contain ring artifacts, resulting from errors produced using the back projection algorithm, which increase with the perpendicular distance from the central slice and the filters used in reconstruction; these enhance frequencies, for example, high frequencies produced by changing sensitivities in adjacent detector elements in the charge coupled device.^45^ This is a systematic error in the reconstruction and does not result in significant errors in the subsequent analysis.

**Figure 6:**
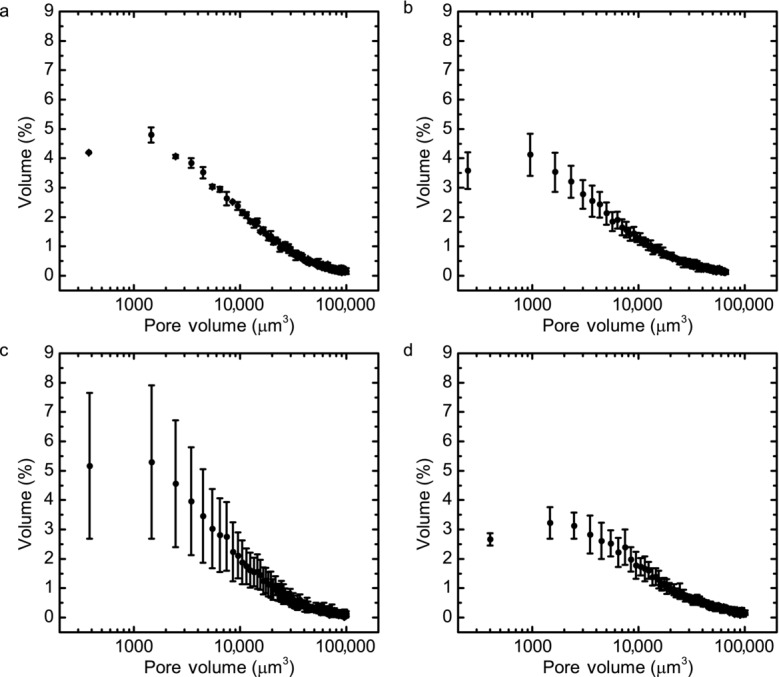
Pore size distributions measured using XμCT. (a) 1.50 mm thick sample of HPMC prepared on the compaction simulator; (b) 1.50 mm thick sample of HPMC prepared using the manual hydraulic press; (c) 1.00 mm thick sample of HPMC prepared using the manual hydraulic press; and (d) 1.50 mm Eudragit sample compressed using the compaction simulator. The error bars represent the SDs between three separate samples analyzed using XμCT.

For further analysis, a region of 512 × 512 pixels at the center of the tablet was used to carry out a quantitative pore size analysis in order to calculate the pore size distribution of the samples, the results of which are shown in Figure 6. Unsurprisingly, the tablets produced using the compaction simulator exhibited a more consistent pore distribution compared with the ones produced using the hydraulic press that reflects the more controlled method of compaction in the compaction simulator that yields a more reproducible microstructure. The highest degree of variation was seen in the 1.0-mm thick HPMC samples.

In all five cases, 4%–5% of the total pore volume are attributed to pore networks with volumes of 1500 μm^3^ or less. In our samples, the HPMC tablets have the largest number of macropores, whereas lactose shows the lowest and is approaching its solid limit at this compaction force^46^ (Table1). Note that typically lactose is compacted at 2–15 kN.^47^

**Table 1:** Bulk Densities, Particle Densities, and Porosities of the Powder Compact Samples

Sample	Mass (g)	Bulk Density (g/cm^3^)	Particle Density (g/cm^3^)	*ξ* (%)	*α* (%)	SD (g/cm^3^)
HPMC (HB)	0.1305	1.11	1.35	5.41	18.3	3.00 × 10^−4^
HPMC (HM)	0.2305	1.16	1.39	5.54	16.6	4.00 × 10^−4^
HPMC (HMT)	0.1331	0.669	1.47	10.1	54.5	1.00 × 10^−4^
Lactose	0.1685	1.43	2.52	3.00 × 10^−4^	43.3	8.32 × 10^−3^
Eudragit	0.1152	0.978	2.03	12.7	43.1	3.42 × 10^−3^

*ξ* is the void space calculated using the XμCT and *α* is the porosity calculated using helium pycnometry. HPMC (HB) are tablets produced using the compaction simulator, HPMC (HM) are tablets that are compacted using the manual hydraulic press, HPMC (HMT) are tablets produced using the manual press with a thickness of 1 mm, lactose refers to tablets that contain lactose, and Eudragit refers to tablets containing only Eudragit RSPO. The standard deviations refer to the bulk densities as measured by helium pycnometry (n=3).

The particle densities (*ρ*_D_) are calculated using the helium pycnometry technique, then using porosity = (1−*ρ*_b_/*ρ*_D_), it is possible to calculate the porosity of the powder compacts. Table1 lists the particle densities, bulk densities, and the porosities calculated using the above equation.

The measured porosities of HPMC, lactose, and Eudragit are in good agreement with literature values.^46^,^48^ It is clear that lactose and Eudragit form more porous structures in comparison to HPMC when using the compaction process chosen for this study.

The porosity and structural data collected are typically used as manufacturing criteria in the pharmaceutical industry, as they are used as an indicator of the drug delivery systems performance. In the future, we aim to link this information to the swelling and diffusion kinetics measured used by TPI, which can be used as a link between microstructure and dissolution studies.

### Analysis of Diffusion and Swelling Kinetics with TPI

**Figure 7:**
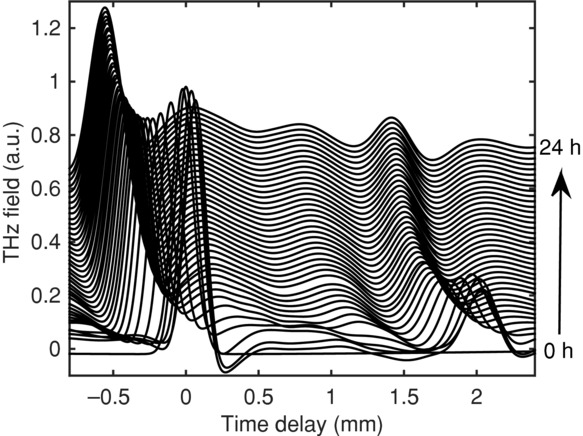
Time–domain waveforms acquired over a period of 24 h during exposure of HPMC to water. Each waveform is offset vertically by 0.0325 a.u. (arbitrary units).

Figure 7 presents a waterfall plot of the time–domain waveforms obtained for the HPMC tablet matrix when in contact with water. The three features discussed in Figure 3 are clearly observed. By following the movement of the front and back surface, the swelling kinetics can be extracted. From the waveforms, it is apparent that the overall tablet thickness remains constant over the first 6 h; for the remainder of the experiment, the tablet dimensions gradually shrink. After 2.5 h, the diffusion front can be resolved, because of a change in refractive index between the hydrated polymer and dry polymer, which is propagating from the wetted tablet surface to the front dry surface.

**Figure 8:**
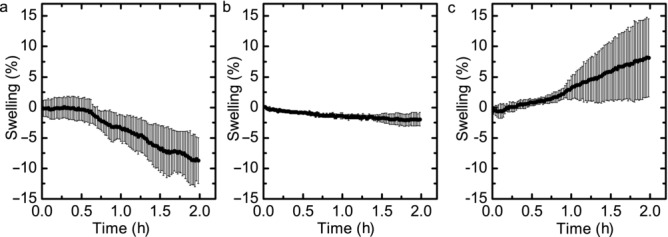
Swelling profiles over a 2.5-h period, where (a) is a 1.5-mm sample of HPMC prepared using a compaction simulator, (b) is a 1.5-mm sample of HPMC prepared on a manual hydraulic press; and (c) is a 1.00-mm HPMC sample also prepared on the manual hydraulic press. The error bars are SDs between three experimental repeats.

Figure 8 shows the initial expansion profiles for the different HPMC samples produced. HPMC samples compressed using the compaction simulator exhibited the largest scale of contraction, where the front face can contract up to 13% of the total tablet thickness (Fig. 8a). The HPMC tablets produced using the manual hydraulic press (Fig. 8b) exhibited very little variation in their geometry, with a total magnitude up to 4% of the total tablet thickness, producing the most reproducible results. The 1.00-mm samples (Fig. 8c) show the greatest variation, with minimal swelling but the largest magnitude of expansion.

One explanation for these changes is the organization of the material within the tablet matrix as discussed by Picker.^36^ The contraction processes observed using the TPI occur over a shorter period and this possibly because of the hydration of the tablet matrices accelerating the process. More work is required to fully understand what the underlying causes of these contractions are. From the experimental data that were acquired from the HMPC sample over 24 h, it was possible to identify a diffusion front in the HPMC matrix. Figure 7 shows the contrast between wetted polymer and dry polymer within the matrix. Following this, response over time can be used to track the diffusion front. This movement can then be related to Eq. (5), which can elucidate the principle control mechanism of the diffusion:


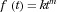
(5)

**Figure 9:**
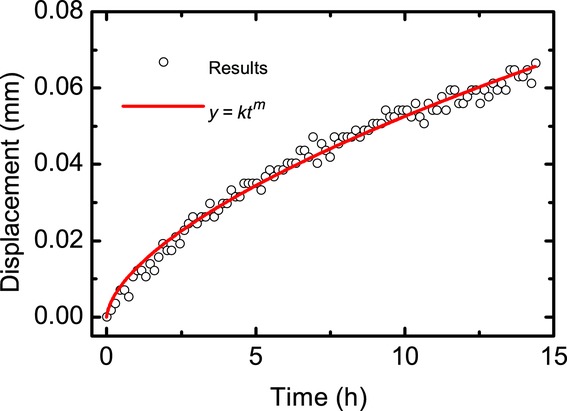
Diffusion front analysis in HPMC; the black points are the diffusion front position at a time *t* and the red lines is fit to Eq. (5).

Figure 9 is the comparison of the experimentally measured diffusion front to theoretical data. When fitting with Eq. (3), *m* is equal to 0.581, with an *R* squared value of 0.983, and root-mean-squared error of 0.006 mm. This *m* value is indicative of anomalous diffusion.^20^

**Figure 10:**
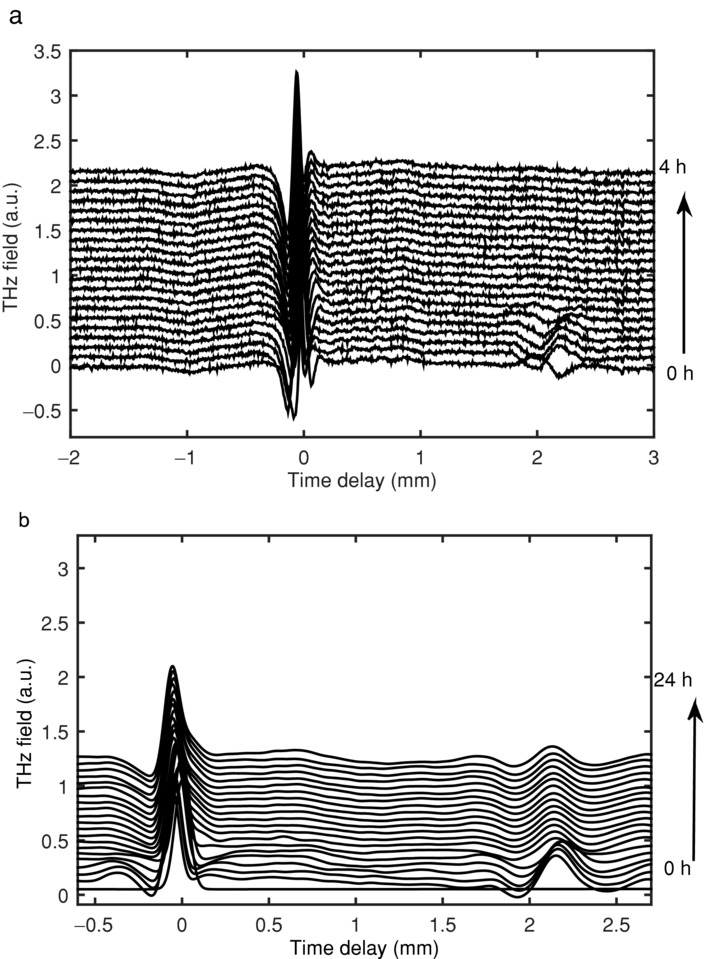
Time–domain waveforms acquired over a period of 4 h during exposure of Eudragit to water. Each waveform is offset vertically by 0.08 a.u. (arbitrary units). (a) A figure of the raw waveforms obtained; (b) the waveforms after deconvolution.

Eudragit is a nonswelling and soluble compound; therefore, when in contact with water it should not swell or disintegrate. Figure 10 shows that there is minimal movement in the Eudragit samples, as expected for this polymer. The matrix is not changing its dimensions and remains stable over a period of 4 h. The use of methacrylate copolymers such as Eudragit has been reported to lack the presence of a swelling and gel forming layer and hence matrices containing these compounds are controlled by erosion^49^ and this is demonstrated in the results obtained using the TPI.

In contrast to the HPMC and Eudragit matrices, the diffusion process is rapid in lactose. The tablet matrix is steadily swelling, until water breaks through the front face of the tablet where a rapid expansion of the tablet geometry occurs. This is expected as lactose is often used as a disintegrant.

**Figure 11:**
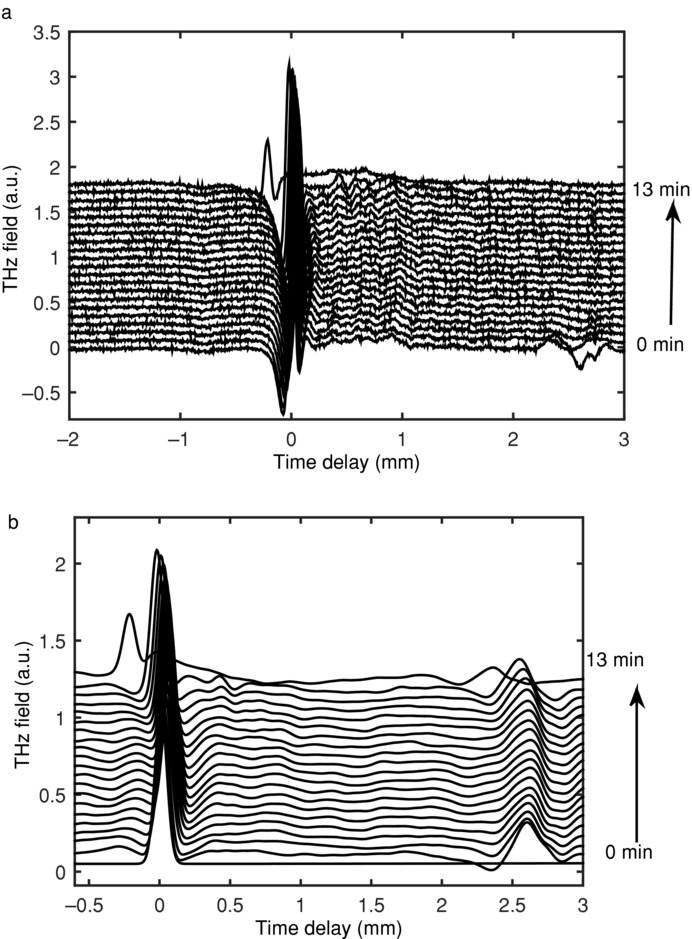
Time–domain waveforms acquired over a period of 13 min during exposure of lactose to water. Each waveform is offset vertically by 0.08 a.u. (arbitrary units). (a) A figure of the raw waveforms obtained; (b) the waveforms after deconvolution.

Figure 11 shows how the lactose waveforms transform over time when in contact with water. When the raw waveforms are deconvolved using the dry tablet as the reference, the high frequency oscillations in the waveforms disappear. After 30 s when in contact with water, these oscillations reappear. The oscillations are a direct consequence of the vibrational features observed in crystalline organic molecular materials.^50^ When lactose tablets are hydrated, they undergo expansion, until they are completely saturated, taking approximately 13 min. At this point, the tablet collapse and the front surface of the tablet rupture, as demonstrated in Figure 11.

## Conclusions

We have demonstrated that terahertz imaging can be used as a powerful tool to investigate diffusion within pharmaceutical powder compacts. It utilizes the contrast between water and polymers to track diffusion while simultaneously assessing the swelling of the polymer matrices.

When in contact with water, the HPMC tablets contract, by approximately 5% of their initial thickness. The tablets then enter a rapid expansion phase where they can expand to up to 20% of their original thickness. Analyzing the diffusion front in HPMC shows that the diffusion of water into the tablets is primarily anomalous diffusion that indicates a mixture of both Fickian diffusion and control from polymer unwrapping. Using the Peppas–Sahlin relation has shown that the bulk of the diffusion is Fickian. Analysis on Eudragits has demonstrated that the matrix remains stable and unchanged when in contact with water; in contrast, lactose shows steady swelling immediately before water breaks through the front face after 13 min. Using pycnometry and X-ray microtomography, these effects were related to the tablet microstructure, which is a step toward understanding how microstructure and compaction affect diffusion in powder compacts.

Experimental data collected in this study have been carried out using a proof of concept apparatus. The set up will be modified to produce more reproducible results, by introducing flow into the set up, to ensure a constant pressure as well as providing a more robust system.

There are still many unanswered questions; the complexity of the swelling kinetics in HPMC requires further study and validation using nuclear magnetic resonance and NIR imaging. Different dissolution mediums will be analyzed including low pH solutions, fasting-state small intestinal fluid, and fed-state small intestinal fluid.
